# Dairy Consumption and Metabolic Health

**DOI:** 10.3390/nu12103040

**Published:** 2020-10-03

**Authors:** Claire M. Timon, Aileen O’Connor, Nupur Bhargava, Eileen R. Gibney, Emma L. Feeney

**Affiliations:** 1 School of Nursing, Psychotherapy and Community Health, Dublin City University, Glasnevin, 9 Dublin, Ireland; claire.timon@dcu.ie; 2 UCD Institute of Food and Health, University College Dublin, Belfield, 4 Dublin, Ireland; aileen.oconnor@ucd.ie (A.O.); nupur.bhargava@ucd.ie (N.B.); emma.feeney@ucd.ie (E.L.F.); 3 School of Agriculture and Food Science, University College Dublin, Belfield, 4 Dublin, Ireland

**Keywords:** dairy, health, matrix, metabolism, nutrient, composition, saturated fats

## Abstract

Milk and dairy foods are naturally rich sources of a wide range of nutrients, and when consumed according to recommended intakes, contribute essential nutrients across all stages of the life cycle. Seminal studies recommendations with respect to intake of saturated fat have been consistent and clear: limit total fat intake to 30% or less of total dietary energy, with a specific recommendation for intake of saturated fat to less than 10% of total dietary energy. However, recent work has re-opened the debate on intake of saturated fat in particular, with suggestions that recommended intakes be considered not at a total fat intake within the diet, but at a food-specific level. A large body of evidence exists examining the impact of dairy consumption on markers of metabolic health, both at a total-dairy-intake level and also at a food-item level, with mixed findings to date. However the evidence suggests that the impact of saturated fat intake on health differs both across food groups and even between foods within the same food group such as dairy. The range of nutrients and bioactive components in milk and dairy foods are found in different levels and are housed within very different food structures. The interaction of the overall food structure and the nutrients describes the concept of the ‘food matrix effect’ which has been well-documented for dairy foods. Studies show that nutrients from different dairy food sources can have different effects on health and for this reason, they should be considered individually rather than grouped as a single food category in epidemiological research. This narrative review examines the current evidence, mainly from randomised controlled trials and meta-analyses, with respect to dairy, milk, yoghurt and cheese on aspects of metabolic health, and summarises some of the potential mechanisms for these findings.

## 1. Contribution of Dairy to a Balanced Diet

**Figure 1:**
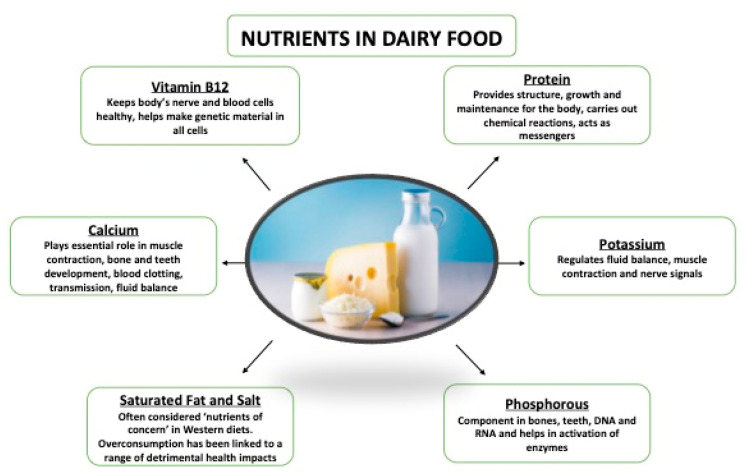
Nutrient content and associated health benefits of dairy consumption.

Milk and dairy foods are naturally rich sources of a wide range of nutrients such as proteins, fats, oligosaccharides and micronutrients including vitamins A, D, E and K and Ca, Mg, P and Zn [1], Figure 1. Milk proteins are of high biological value, not only because they contain essential amino acids but also because of their high digestibility and bioavailability. Approximately 80% of milk protein is casein and the remaining 20% is serum, or whey protein for cow’s milk [2]. Fat, mainly in the form of triacylglycerols (98%), is present in milk as globules which are surrounded by a membrane (or milk fat globule membrane (MFGM)). This component of milk fat has been suggested to elicit favourable lipid and low-density lipoprotein (LDL) cholesterol response to dairy consumption [3], and will be discussed later. With respect to micronutrients, milk is considered a major source of calcium in the diet [4]. As well as being a rich source of this nutrient, the bioavailability of calcium from dairy sources has also been shown to be higher compared to other dietary sources [5,6]. Furthermore, modelling of dietary intake data has indicated that, without consuming dairy products, less than half of the dietary calcium requirements would be met [7]. The authors of that study also noted that nutrients from dairy foods are difficult to replace and modelled removal and replacement with available alternatives, which resulted in lower amounts of several nutrients including protein, phosphorus, riboflavin, zinc and vitamin B12 [7]. While there is some concern that avoidance of dairy may have implications for some nutrients, this does not suggest that adequate nutrient intake from a low-dairy or dairy-free diet is unattainable, but rather indicates that dairy can significantly contribute towards a healthy diet.

When consumed according to recommended intakes of national guidelines, milk and dairy products contribute essential nutrients across all stages of the life cycle [1]. For example, milk and dairy products are an important part of a young child’s diet as they are a good source of energy and protein and contain a wide range of vitamins and minerals, especially calcium, that young children need for healthy bones and teeth [8]. In Europe, it is reported that milk contributes proportionally more to the diets of young children than to adults [9,10]. Data from cross-sectional studies [11] and intervention studies [12] have reported the positive effect of milk consumption in childhood and adolescence on bone mineral content and bone mineral density. In addition, some research studies have indicated that the consumption of milk and milk products during adolescence is associated with neutral or reduced risk of adiposity [13,14]. During pregnancy, dairy products can be an important means of providing adequate calcium and other key nutrients in the diet [15]. Evidence from prospective cohort studies suggests that moderate milk consumption compared to none or low intakes during pregnancy is positively associated with foetal growth and infant birth weight in healthy, Western populations [16]. Finally, several studies point to the benefits of milk and dairy products in diets of the elderly and highlight that, in combination with physical activity, milk and dairy products can improve muscle mass and function resulting in a lower risk of sarcopenia and vertebral fractures [16], although another recent review of the area did not find strong evidence for a benefit of milk on muscle health in older adults [17].

Current consumption patterns of dairy are in a period of considerable change, with reported decreases in dairy consumption in countries who have traditionally consumed large quantities, potentially due to increased intake of ‘dairy-free’ alternative products [18] and reported increases in some global regions, where dairy has not been commonly consumed [19]. These changes are reported to be due to several factors, but are predominantly driven by consumer perception of the health effects of dairy consumption and the environmental impact of dairy production [20]. Particular nutrients of concern, when considering intake of dairy, are sodium and fat. Whilst dairy has been shown to contribute beneficially to the diet, dairy foods also contribute significantly to sodium and saturated fat intakes (Figure 1). High dietary salt intake is a prominent factor for the development of hypertension, a strong risk factor for cardiovascular disease [21,22]. In the US and UK, dairy products are significant contributors to dietary salt intakes, providing approximately 11% and 8% of overall intakes, respectively [23,24]. Dietary efforts to reduce hypertension include the well-established DASH diet (Dietary Approaches to Stop Hypertension), where low-fat dairy products are important characteristics [25]. In relation to dairy fat, the 2011–2014 National Health and Nutrition Examination Survey (NHANES) demonstrated that dairy foods contributed 26% of saturated fat and 14.2% of total fat to the diets of US adults [26].

Similarly, Feeney et al. observed that, within the Irish diet, dairy foods contributed 12.8% of total fat to the diet, and 19.8% of the saturated fat [27]. For this reason, many healthy eating guidelines recommend 3–5 portions of dairy daily, with consumption of “low/reduced-fat dairy” when possible [28,29]. However, research investigating the importance of the food source of saturated fatty acids (SFA) suggests that although SFAs from meat and processed-meat are associated with detrimental health effects [30], SFA intake from dairy sources may be associated with either neutral [31] or beneficial effects on cardiovascular health markers [32,33]. Further, the individual dairy sources may have different impacts. Much work in this area is underway, which is summarised below.

## 2. Dairy Fat and the Link to Health

Since the seminal Seven Countries Study and other subsequent studies [34,35], recommendations with respect to intake of saturated fat have been consistent and clear: limit total fat intake to 30% or less of total dietary energy, with a specific recommendation for intake of saturated fat to less than 10% of total dietary energy [36,37,38]. However, recent work has re-opened the debate on intake of saturated fat in particular [39]. In a large review and meta-analysis, de Souza et al. examined associations between intake of total fat, saturated fat and trans-unsaturated fat with all-cause mortality and differing morbidities. The authors concluded that, contrary to previous evidence, saturated fat intake was not associated with all-cause mortality, cardiovascular disease (CVD) mortality, total coronary heart disease (CHD), ischemic stroke or type 2 diabetes [39]. Drouin-Chartier et al. in 2016 also examined the impact of dairy consumption and dairy fat on cardiometabolic disease risk factors, and also reported that the purported detrimental effects of SFAs on cardiometabolic health may in fact be nullified when they are consumed as part of complex food matrices such as those in cheese and other dairy foods [40]. Similarly, Alexander et al. (2016) completed a meta-analysis of prospective studies and the intake of dairy products and CVD risk. These authors more cautiously concluded that although for some individual dairy products risk estimates below 1.0 were observed, additional data are needed to more comprehensively examine potential dose–response patterns [41].

### 2.1. Dietary Guidelines—Nutrient-Based vs. Food-Based

Despite these more recent findings, the most recent review of published literature by the UK Scientific Advisory Committee on Nutrition (SACN) concluded that there is a significant body of evidence demonstrating a relationship between intake of saturated fats and CVD and CHD events, but not CVD and CHD mortality [28], and noted that, irrespective of the lack of evidence for an effect on mortality, non-fatal CVD and CHD events have a serious adverse impact on health and quality of life, and that existing public health recommendations for saturated fat to be <10% of total dietary energy intake were still valid [42]. This mirrors recommendations in other countries and regions of the world [36,37,38]. However, some criticisms on the continued support for such recommendations note that such policies are based on evidence from total dietary saturated fat intake, and may have not considered the source of fat, whereby the food source, or matrix may in fact have a differing influence on metabolism and subsequently, health [43]. In addition, different food sources contain different types and amounts of SFA, and the continued promotion of an overall <10% of total dietary energy recommendation may be perceived to overlook this [43]. Further, by focusing on SFA content alone, foods that are nutrient-dense but also high in SFA may be excluded from the diet and inadvertently result in a reduced intake of important micronutrients [43]. For this reason, many are advocating for food-based guidelines rather than nutrient-based advice. As such, evidence may need to be considered at a food-item level within the dairy food group, rather than together. In light of a reported shift in recent dairy consumption [19], where instances of decreased dairy consumption may be potentially explained by an increase in dairy-free alternatives due to the perceived impact of dairy on health [18], it is important to consider some of the recent evidence at both a total-dairy level and for individual dairy products cheese, milk and yoghurt, and then further discuss potential mechanisms influencing the metabolic response to consumption.

### 2.2. Total Dairy vs. Specific Products

Dehghan et al. (2018) specifically examined the associations between total dairy and specific types of dairy products with mortality and major cardiovascular disease. Dietary intakes of dairy products for 136,384 individuals were recorded using country-specific validated food frequency questionnaires and associations with mortality or major cardiovascular events were examined [44]. The authors reported that a higher intake of total dairy (>2 servings per day compared with no intake) was associated with a lower risk of the composite of mortality or major cardiovascular events, total mortality, non-cardiovascular mortality, cardiovascular mortality, major cardiovascular disease and stroke. No significant association with myocardial infarction was observed. Higher intake (>1 serving vs. no intake) of milk and yoghurt was associated with lower risk of the composite outcome, whereas cheese intake was not. Butter intake was low and was not significantly associated with clinical outcomes. The authors concluded that dairy consumption was associated with lower risk of mortality and major cardiovascular disease events in a diverse multinational cohort [44].

Fontecha and colleagues [45] specifically examined evidence regarding the influence of dairy product consumption on the risk of major cardiovascular-related outcomes and how various doses of different dairy products affected such responses. In this overview of 12 meta-analyses involving randomised controlled trials (RCTs), as well as the updated meta-analyses of RCTs, increasing consumption of dairy products did not result in significant changes of known risk biomarkers such as systolic and diastolic blood pressure and total cholesterol and LDL cholesterol. They concluded that consumption of total dairy products (either regular or low-fat content), did not adversely affect the risk of CVD [45].

Considering total dairy consumption initially, characteristics of fifteen published RCTs that studied the effects of overall dairy consumption on markers of metabolic health and CVD risk on variable age groups are included in Table 1. RCTs included in the table were either parallel or crossover trials and the participants’ ages ranged from 20 to 75 years. Duration of interventions varied widely from 4 to 8 weeks in some to 12 to 24 weeks in the others, with a washout period included in some of the trials. Most of the interventions were based on consumption of different dairy products such as milk, cheese or butter. Zemel et al. (2010) compared the effects of consumption of soy-based smoothie to a dairy-based smoothie and observed suppressed inflammatory and stress markers for the latter [46]. Groups that consumed a low-fat or non-fat dairy diet showed decreased levels of total cholesterol (TC) and low-density cholesterol (LDL) as compared to the diet containing conventional levels of fat. Most recently, Vasilopoulou et al. (2020) examined the impact of modified dairy fat consumption, through the provision of products with modified monounsaturated fatty acid (MUFA) content in adults at moderate CVD risk—the RESET study (controlled REplacement of SaturatEd fat in dairy on Total cholesterol). The authors concluded that consumption of a high-fat diet containing modified dairy products with reduced saturated fatty acids, and enriched monounsaturated fatty acids showed beneficial effects on fasting LDL cholesterol and endothelial function compared with conventional dairy products [47]. Finally, although not an intervention study, Drouin-Chartier (2019), recently examined dairy intake and risk of diabetes, and reported positive impacts for yoghurt and reduced-fat milk, but a negative association for cheese [48].

### 2.3. Cheese

Focusing specifically on cheese, several published RCTs have demonstrated a beneficial effect of cheese consumption on markers of metabolic health and CVD risk, summarised in Table 2. Brassard et al. (2017) compared the impact of consuming equal amounts of SFAs from cheese and butter on cardiometabolic risk factors [61]. In this multicentre, crossover, randomised controlled trial, participants were assigned to a randomised sequence of five isoenergetic diets of 4-week duration (separated by 4-week washout periods). The diets were rich in SFAs from either cheese or butter, or a monounsaturated fatty acid (MUFA)–rich diet, a polyunsaturated fatty acid (PUFA)–rich diet and a low-fat, high-carbohydrate diet. The authors reported that serum HDL-cholesterol concentrations were similar after the cheese and butter diets but were significantly higher in comparison to response after the carbohydrate diet. Comparing cheese and butter, LDL-cholesterol concentrations after the cheese diet were lower than after the butter diet but were higher than after all of the other diets. Some variation in response was noted. Work conducted by this research group previously has both supported and added to the existing evidence [32]. A 6-week randomised parallel intervention involving 164 volunteers who received ~40 g of dairy fat/d, in 1 of 4 treatments: 120 g full-fat Irish cheddar cheese (group A), 120 g reduced-fat Irish cheddar cheese + butter (21 g) (group B); butter (49 g), calcium caseinate powder (30 g) and Ca supplement (CaCO_3_) (500 mg) (group C) or 120 g full-fat Irish cheddar cheese, for 6 weeks following completion of a 6-week “run-in” period, where this group excluded all dietary cheese before commencing the intervention (group D). This study found that a stepwise-matrix effect was observed between the groups for total cholesterol (TC) (P = 0.033) and LDL cholesterol (P = 0.026), with significantly lower post-intervention TC and LDL cholesterol when all of the fat was contained within the cheese matrix (Group A), compared with Group C when it was not. These findings suggest that dairy fat, when eaten in the form of cheese, appears to differently affect blood lipids compared with the same constituents eaten in different matrices, with significantly lower total cholesterol observed when all nutrients are consumed within a cheese matrix. This ‘dairy matrix’ concept, whereby the nutrients within a dairy food interact with the overall structure providing different health effects, is becoming increasingly studied, [62], and is discussed further in Section 3, below.

Other groups have also looked at postprandial response to cheese consumption, as the postprandial response to lipid consumption is considered an independent indicator of CVD risk [75]. Drouin-Chartier (2017) reported minor differences in postcirculating TAG concentrations, in a postprandial RCT where participants ingested 33 g fat from a firm cheese (young cheddar), a soft cream cheese (cream cheese) or butter (control) incorporated into standardised macronutrient-matched meals. They conclude that the study demonstrates that the cheese matrix modulates the impact of dairy fat on postprandial lipemia in healthy subjects [75].

Hansson et al. 2019, examining postprandial response to sour cream, whipped cream, cheese and butter, noted that sour cream resulted in a larger postprandial triacylglycerol (TAG) area under the curve (AUC), compared to whipped cream, butter and cheese (P = 0.05). Intake of sour cream also induced a larger HDL cholesterol AUC compared to cheese. Intake of cheese induced a 124% larger insulin AUC compared to butter. Hansson et al. concluded that high-fat meals containing similar amount of fat from different dairy products induce different postprandial effects on serum TAGs, HDL cholesterol and insulin in healthy adults [76].

### 2.4. Milk

Characteristics of seven RCTs that studied the effects of milk consumption on markers of metabolic health and CVD risk are described in Table 3 [77,78,79,80,81,82,83]. RCTs included in the table were either parallel or crossover trials and the participants’ ages ranged from 20–85 years. Duration of interventions ranged from 4 to 16 weeks with a washout period included in some of the studies. Gardner et al. (2007) compared the effects of soy-based drinks to dairy milk consumption on metabolic health markers, and found that LDL was significantly lower after consuming soy milk compared to dairy milk, but no significant differences between groups were observed for HDL, triacylglycerols, insulin or glucose [77]. In 2016, Lee et al. examined the impact of milk consumption (400 mL per day) compared to habitual intake on markers of metabolic health, and found no significant differences in body mass index, blood pressure or lipid profile [78]. Hidaka et al. compared intakes of full-fat vs. non-fat milk intakes and showed lower plasma triglyceride and phospholipid levels in the no-fat group [79].

### 2.5. Yoghurt

Table 4 presents a summary of RCTs that specifically studied the effects of yoghurt consumption on markers of metabolic health and CVD risk. Most of these studies focused specifically on the microbial content of yoghurts, where consumption of probiotic yoghurt or modified-bacterial-strain-containing yoghurts or comparisons between commonly available varieties of yoghurts such as non-fat yoghurt, natural yoghurt and heated yoghurt were examined. In general, when compared to participants on a controlled diet (diet with zero consumption of fermented products), the participants who consumed probiotic or conventional yoghurt showed significant decrease in TC and LDL cholesterol [84,85,86,87,88,89,90,91,92].

## 3. The ‘Dairy Matrix’

**Figure 2:**
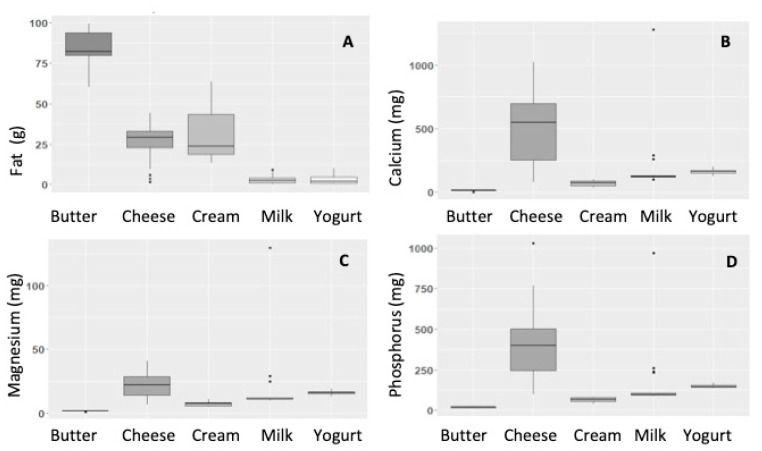
Boxplot showing average fat and mineral content in dairy products per 100 g. Values from the Composition of Foods Integrated Dataset (CoFID). Only plain, unflavoured products with no added sugar were included in this analysis. Nutrients shown are: (**A**) total fat (g), (**B**) calcium (mg), (**C**) magnesium (mg) and (**D**) phosphorus (mg), calculated from *n* = 4 butter, *n* = 43 cheese, *n* = 8 cream, *n* = 20 milk and *n* = 4 yoghurt samples.

While dairy products are often considered together as a food category in nutritional epidemiology, they vary considerably in terms of their content and structure and how these interact with other food components, which describes the ‘dairy matrix’ concept [93]. Values from the Composition of Foods Integrated Dataset (CoFID) were used to compare nutrient composition across the range of commonly available dairy products (summarised in Figure 2). While they represent a wide range of products, only plain, unflavoured versions with no added sugar were included in this analysis. Figure 1 demonstrates how nutrient composition across the range of products varies greatly, as does their overall matrix or structure, depending on the product type. For example, whole milk contains 3.6% fat in a liquid oil-in-water emulsion with lactose and protein (both casein and whey) while cheeses mainly consist of casein proteins and fat, in a solid matrix, with only trace levels of lactose and whey [62]. Butter is an emulsion of water-in oil, and contains mostly fat and water, with no protein or carbohydrate, while (liquid) cream is also a water-in-oil emulsion, and contains low levels of protein (approx. 2%) [62] and lactose (approx. 3%) (in 35%-fat cream). The various processing steps that different products undergo from raw milk to final foodstuff impact the level of the different nutrients and the overall macro and microstructure of these foods. The nature of these differences may result in the different health outcomes associated with their consumption.

Cheese in particular is associated with lower levels of blood cholesterol than other dairy products and especially when compared to butter (see Table 2 for an overview of studies in this area). Cheese structures contain aggregated casein micelles [94] which may impact the ability of lipases to break down the fat contained within the matrix, compared to the same fat contained within other food matrices (e.g., milk and butter). The structure of cheese, including the degree of hardness and cohesiveness, can result in it being more physically resistant to digestion than other matrices [94] which affects the degree to which the fat can be digested and absorbed. There may be additional effects from the calcium contained within this matrix, reacting with the fatty acids to form insoluble calcium soaps [95], as well as the separate textural effects from calcium that increase the cohesiveness [94] which may result in enhanced digestive resistance. This mechanism also appears to be supported by a higher faecal fat excretion following cheese consumption compared to other sources, in postprandial studies [67] although is not fully confirmed [68]. In addition to the calcium content of cheese, the phosphorus content is also implicated in the reduction of fat digestibility, by affecting the ability of cheese constituents to form insoluble soaps during digestion (calcium phosphate). This is thought to further increase fat excretion via the adsorption of bile acids to the surface, and has been implicated in the reduction of LDL-c [62,95]. Both phosphorus and calcium are particularly concentrated in cheese compared to other dairy products [96].

Cheese is a fermented dairy product, and the fermentation process may also be one of the contributors to the cardiometabolic protective nature of cheese via a number of mechanisms. Lactic acid bacteria found in fermented dairy products can result in platelet-activating factor (PAF)-inhibitory lipid production [97,98]. Further, as cheeses ripen and age, shorter peptides are produced and in some cases there is a release of latent bioactives, as some of these peptides have specific bioactivity that is not apparent in the intact ‘parent’ protein [99,100]. In cheeses, antihypertensive peptides are produced during fermentation, including V-P-P and I-P-P, which are tripeptides that exert their effects via inhibition of the angiotension converting enzyme (ACE) pathway [101,102]. Some cheeses have also been found to have bioactivity related to glycaemic control [103], which may also contribute to the cardio-metabolic benefits from cheese consumption. The starter culture used in the cheesemaking process can have an additional impact on the inherent bioactivity produced during fermentation [104,105]. Finally, the form in which fat is contained in cheese compared to butter may also result in some of the differences observed between these two products. The polar lipids found in dairy products in general appear to have anti-inflammatory properties compared to other oxidised dietary lipids [106,107,108] and they are mostly contained in a bioactive envelope surrounding the fat, known as the milk fat globule membrane (MFGM) [109]. Cheese contains particularly high levels of polar sphingolipids that are not present in the same levels in butter, since the membrane is disrupted during the churning process [62,110]. Studies suggest that polar lipids can impact blood lipid levels in the acute postprandial period, with lower lipaema (and insulin) observed following a liquid meal of palm fat when an MGFM-rich dairy fraction was added, compared to the same meal without this addition [111].

With research strongly suggestive of postprandial hyperlipidema as an independent risk factor for CVD [112], this could be a further explanation for the growing list of studies showing a protective effect of cheese consumption on CVD risk [32,60,61,68,72,113,114,115] despite the relatively high SFA content.

## 4. Conclusions/Future Directions

This paper summarises and discusses the evidence that examines the link between dairy intake and CVD risk. Whilst the evidence is mixed for some dairy foods (milk), it is more consistent for others (cheese/yoghurt), and supports the concept that the source of saturated fat intake has an important impact on cardiometabolic response to consumption. This is not a new concept, but whilst this evidence is growing, more research is needed before any significant change in public health recommendations are implemented.

While the link between dairy foods and metabolic health has been well-studied, research gaps still remain, and must be considered in future work. Many of the RCTs completed are short-term studies with single products, and there is a need to consider combinations of foods in a dietary pattern, considered by only a few studies to date [116,117]. The manner of the food consumption also needs to be considered, as dairy is often consumed in many forms (heated, melted) and as part of meals or recipes or in sweetened beverages. In addition, the amount of dairy product given in many of the randomised controlled trial studies to date are largely outside of recommended portion size intakes, as such, caution is required in interpreting and generalising findings.

Looking to the future, to date many of the studies have focused on traditional markers of cardiometabolic risk, particularly circulating lipid levels, which may not show subtle changes between products and/or further elucidate the cause/mechanisms for difference in response following consumption of specific foods. It has been suggested that the use of such traditional markers alone may limit the ability to predict health outcomes from the fat in dairy products, since many other components in dairy may have impact on CVD risk [118]. For this reason, more recent studies are also examining novel biomarkers that include vascular function (arterial stiffness, flow-mediated vasodilation (FMD) [47] and LDL-c particle size distribution [119]. It is important that we more fully understand the mechanisms underlying the variance in response to consumption, and the use of such novel markers will help develop this knowledge.

In conclusion, dairy foods are diverse in their structure and their nutrient content, resulting in differing biological responses and associated health outcomes. Thus, continuing to treat them as a single food category in food-based healthy eating guidelines may obscure the individual effects of these foods. As diets transition, there is an urgent need to understand the impact of different dairy foods, their preparation methods and how they are consumed, within the overall patterns of dietary intake in different cultural groups.

## Figures and Tables

**Table 1:** Randomised controlled trials (RCT) demonstrating an effect of overall dairy consumption on markers of metabolic health and cardiovascular disease (CVD) risk.

Author	Country	Study Design	Population	Age (Years)	Intervention	Duration	Main Findings
Vasilopoulou et al. 2020 [47]	UK	Crossover	*n* = 54 (31 male; 23 female), with risk of CVD	25–70	2 arm: **(A)** monounsaturated fatty acid (MUFA)-modified dairy—340 g UHT milk, 45 g cheese, 25.1 g butter. **(B)** Control—340 g ultrahigh temperature (UHT) pasteurised milk, 45 g cheese, 25.1 g butter	Two 12-week periods separated by an 8-week washout period	No significant change from baseline in serum total cholesterol (TC) between diets. Group **A** had a significant beneficial effect in terms of attenuation of the rise of the low-density lipoprotein (LDL) cholesterol. No changes in high-density lipoprotein (HDL) cholesterol between diets. The LDL:HDL ratio decreased significantly after group **A**, and increased after the control. No significant differences were observed for indexes of insulin sensitivity/resistance. Fasting plasma nitrite concentrations increased after the modified diet, yet decreased after the control.
Markey et al. 2017 [49]	UK	Crossover	*n* = 54 (31 male; 23 female), with risk of CVD	25–70	2 arm: **(A)** MUFA-modified dairy—340 g UHT milk, 45 g cheese, 25.1 g butter. **(B)** Control—340 g UHT milk, 45 g cheese, 25.1 g butter.	Two 12-week periods, separated by an 8-week washout period	Group **A** showed a smaller increase in saturated fatty acids (SFA) and greater increase in MUFA intake when compared with the control.
Rosqvist et al. 2015 [3]	Sweden	Parallel	*n* = 57 (gender split not stated), overweight or obese	20–70	2 arm: **(A)** milk-fat globule membrane (MFGM) group—100 mL whipping cream (40%fat)/d, 100 mL fat-free milk (0.1% fat)/d and 1 scone/d (baked with wheat flour, water, sodium chloride and baking powder). **(B)** Fat-free milk, 100 mL, (0.1% fat)/d and 1 scone/d (baked with wheat flour, water, butter oil (98.7% fat), sodium chloride, baking powder and milk protein isolate).	8 weeks	Control diet increased TC, LDL, apolipoprotein B:apolipoprotein A-I ratio and non-HDL plasma lipids, whereas the MFGM diet did not. HDL, triglyceride, sitosterol, lathosterol, campesterol and proprotein convertase subtilisin/kexin type 9 concentrations and fatty acid compositions did not differ between groups.
Benatar et al. 2013 [50]	New Zealand	Parallel	*n* = 180 (54 male; 126 female), healthy volunteers	>18	3 arm: **(A)** increased dairy—an extra two-to-three servings per day, and to change to high-fat milk and dairy solids. **(B)** Habitual dairy intake remains unchanged. **(C)** Decreased dairy were asked to eliminate all possible sources of dairy.	1 month	No significant change in LDL or HDL, triglycerides, systolic or diastolic BP, C-reactive protein, glucose or insulin across groups. There was a small increase in weight in group **A**.
Nestel et al. 2013 [51]	Australia	Crossover	*n* = 12 (gender not specified) overweight or obese	40–70	3 arm: **(A)** low-fat dairy, 1% fat milk (400 mL/d) and 1% fat yoghurt (200 g/d). **(B)** Full-fat dairy (fermented), cheddar cheese (85 g/d) and full-cream yoghurt (three servings, 600 g/d). **(C)** Full-fat dairy (non-fermented), butter (30 g/d) and cream (70 mL/d) and small amounts of ice-cream.	Two 3-week periods, for group B + C (full fat diets). Group A diet (low fat) was consumed twice—between and at the end of the full-fat dairy dietary periods, for a duration of 2 weeks.	Lowest LDL and HDL concentrations were observed in group **A**, but plasma Triacylglycerol (TAG) concentrations did not differ significantly across the groups. Concentrations of plasma sphingomyelin and IL-6 were significantly higher after the non-fermented dairy diet (group **C**) than the low-fat dairy diet
Crichton et al. 2012 [52]	Australia	Crossover	*n* = 61 (18 male; 43 female), overweight or obese	18–75	2 arm: **(A)** 4 servings of reduced-fat dairy/d, 1 serving = 250 mL milk, 175–200 g yoghurt and 190 g custard. **(B)** Control—1 serving of dairy/d, reflecting habitual intake.	Two 6-month periods, no washout period	No significant changes in resting metabolic rate or total energy expenditure, systolic and diastolic BP, fasting blood glucose, TC, HDL or LDL, triglycerides or *hs*-CRP (high sensitivity C-reactive protein). Additionally no differences between groups for waist circumference (WC), body weight and fat mass.
Palacios et al. 2011 [53]	Puerto Rico	Parallel	*n* = 25 (5 male; 20 female), obese	22–50	3 arm: **(A)** 4 servings of dairy/d (low-fat milk, low-fat cheese and low-fat yoghurt), with a dairy-calcium intake goal of 1200–1300 mg/d. **(B)** calcium supplement (600 mg/d, calcium carbonate). **(C)** Control—habitual diet with placebo tablet.	21 weeks	No significant group effects were observed for anthropometric measurements or serum lipids such as TC, HDL, LDL and TAG levels. Although TAG levels decreased by 18% in group **A** (high dairy).
Stancliffe et al. 2011 [54]	USA	Parallel	*n* = 40 (19 male; 21 female), overweight or obese with metabolic syndrome	37.0 ± 9.9	2 arm: **(A)** low-dairy diet—0.5 servings/d and provided with 3 servings/d of non-dairy foods that is low-sodium luncheon meats, soy-based luncheon meat substitutes, packaged fruit cups, granola bars and peanut butter crackers. **(B)** adequate dairy diet—3.5 servings/d, of which 2/3 servings were milk and/or yoghurt.	12 weeks	Group **A** decreased malondialdehyde and oxidised LDL. Inflammatory markers were suppressed with intake of AD, with decreases in TNF-a (Tumor Necrosis Factor-a); decreases in IL-6 (Interleukin-6) and monocyte chemoattractant protein 1 and an increase in adiponectin. Group **B** exerted no effect on oxidative or inflammatory markers. Group **A** significantly reduced waist circumference and trunk fat but group **B** exerted no effects.
van Meijl and Mensink, 2010 [55]	Netherlands	Crossover	*n* = 35 (10 male; 25 female), overweight or obese	18–70	2 arm: **(A)** 500 mL low-fat milk and 150 g low-fat yoghurt per day. **(B)** Control—600 mL fruit juice and 43 g fruit biscuits per day	Two 8-week periods separated by a 2-week washout period	Plasma concentrations of TNF-a decreased, and soluble TNF-a receptor-1 increased after low-fat dairy consumption compared to the control. s-TNFR-2 also increased. Low-fat dairy consumption had no effect on IL-6, monocyte chemoattractant protein-1, intracellular adhesion molecule-1 and vascular cell adhesion molecule-1 concentrations. Lipid profiles were not analysed.
Zemel et al. 2010 [46]	USA	Crossover	*n* = 20 (14 male; 6 female), overweight or obese	Average—31 ± 10.3	2 arm: **(A)** dairy smoothie, 3 times/d, with non-fat dry milk as the protein source, and containing 350 mg calcium per smoothie. **(B)** Soy smoothie, 3 times/d, with soy protein isolate as the protein source and 50 mg calcium per smoothie.	Two 4-week periods separated by a 4-week washout period	Group **A** resulted in significant suppression of oxidative stress and lower inflammatory markers; tumour necrosis factor-a; IL-6; monocyte chemoattractant protein-1 and increased adiponectin. Group **B** exerted no significant effects. Lipid profiles were not analysed.
Wennersberg et al. 2009 [56]	Norway	Parallel	*n* = 121 (41 male; 80 female)	30–65	2 arm: **(A)** milk group, 3–5 portions of dairy/d. Portion = 200 g milk, 200–250 g yoghurt or sour milk, 75 g cream or crème fraıche, 15–40 g cheese, 3–10 g butter or butter-containing spreads, 50 mL cottage cheese, and ice-cream occasionally. **(B)** Control—habitual daily diet.	6 months	No significant differences between changes in body weight or body composition, BP, markers of inflammation, endothelial function, adiponectin or oxidative stress in group **A** and **B**. There was a modest unfavourable increase in serum TC concentrations in the group **A**.
van Meijl and Mensink, 2009 [57]	Netherlands	Crossover	*n* = 35 (10 males; 25 females), overweight or obese	18–70	2 arm: **(A)** 500 mL low-fat milk and 150 g low-fat yoghurt per day. **(B)** Control—600 mL fruit juice and 43 g fruit biscuits per day	Two 8-week periods separated by a 2-week washout period	In group **A**, systolic BP significantly decreased compared with the control, but diastolic BP did not reach significance. Decreases in HDL and apo A-1 concentrations were also observed in group **A**. Serum TC, LDL, apo B, TAG, non-esterified fatty acids, glucose, insulin, C-reactive protein and plasminogen activator inhibitor-1 remained unchanged.
Tricon et al. 2006 [58]	UK	Crossover	*n* = 32 males, healthy volunteers	34–60	2 arm: **(A)** 500 mL UHT full-fat milk, 12.5 g butter and 36.3 g cheese per day, naturally enriched with CLA (conjugated linoleic acid). **(B)** Control, 500 mL UHT full-fat milk, 12.5 g butter and 28 g cheese per day.	Two 6 week periods, separated by a 7 week washout period	Diet **A** did not significantly affect body weight, inflammatory markers, insulin, glucose, TAG, or TC, LDL and HDL cholesterol but resulted in a small increase in the LDL:HDL ratio. The modified dairy products changed LDL fatty acid composition but had no significant effect on LDL particle size or the susceptibility of LDL to oxidation.
Zemel et al. 2005 [59]	America	Parallel	**Study 1.***n* = 34 (11 male; 23 female), **Study 2.** *n* = 29 (4 male; 25 female), obese	26–55	**Study 1. (A)** Dairy group—3 servings of dairy/d, at least one serving to be milk **(B)** Control—low dairy, 0–1 servings of low-fat dairy/d. **Study 2.** **(A)** Dairy group—3 servings of dairy/d, at least one serving to be milk and a 500-kcal/d deficit **(B)** Control—low dairy, 0–1 servings of low-fat dairy/d and 500 kcal/d deficit diet.	24 weeks (both studies)	**Study 1.** Body weight remained stable for both groups. Group **A** resulted in decreases in total body fat, trunk fat, insulin and BP and an increase in lean mass and no significant changes in the control group. **Study 2.** Both diets produced significant weight and fat loss. Weight and fat loss within group **A** were 2-fold higher, and loss of lean body mass significantly reduced compared with the control. There were no effects on circulating lipids in either group.
Tholstrup et al. 2004 [60]	Denmark	Crossover	*n* = 14 males, healthy volunteers	20–31	3 arms: **(A)** 1.5 L of whole milk/10 MJ (54 g of fat and 1779 mg calcium per 10 MJ). **(B)** Butter 64 g/10 MJ (54 g of fat and 10 mg calcium per 10 MJ) **(C)** 205 g of hard cheese, “Samsø”, 45% fat of dry weight, i.e., 26% fat/10 MJ (1989 mg calcium/10 MJ).	Three 3-week periods separated by a 4-week washout period	Fasting LDL concentrations were significantly higher after butter than cheese diet, with a borderline significant difference in TC after the experimental periods. Postprandial glucose showed a higher response after cheese diet compared to milk diet. No differences were found between groups for HDL, Very Low-Density Lipoprotein (VLDL), apo A-1 and apo B concentrations.

**Table 2:** Randomised controlled trials (RCT) demonstrating an effect of cheese consumption on markers of metabolic health and CVD risk.

Author	Country	Study Design	Population	Age (Years)	Intervention	Duration	Main Findings
Feeney et al. 2018 [32]	Ireland	Parallel	*n* = 164 (75 male; 89 female), BMI > 25 kg/m^2^	>50	4 arm: **(A)** 120 g full-fat cheddar cheese (FFCC). **(B)** Reduced-fat Irish cheddar cheese, 120 g, +butter (21 g) (RFC + B). **(C)** Butter (49 g), calcium caseinate powder (30 g), Ca supplement (BCC). **(D)** Full-fat Irish cheddar cheese, 120 g, (as per “a” but with 6-week run-in period).	6 weeks	There was a significant difference in total cholesterol (TC) and LDL between groups. Group **A** had significantly lower TC and LDL compared with other groups. No differences were observed for HDL cholesterol, anthropometry, fasting glucose or insulin.
Limongi et al. 2018 [63]	Italy	Crossover	*n* = 58 (16 male; 42 female), healthy volunteers	>60	2 arm: **(A)** 90 g/d of CLA-enriched Pecorino cheese. **(B)** Control, 90 g/d of Pecorino cheese.	Two 2-month periods, separated by 1-month washout period	No significant differences found in relation to LDL between diet **A** + **B**. Participants consuming enriched cheese had a lower increase in glycaemia compared to control but did not display an increase in lipid levels.
Brassard et al. 2017 [61]	Canada	Crossover	*n* = 92 (43 male; 49 female), abdominally obese	18–65	5 arm: **(A)** 90 g/2500 kcal cheese (type not specified). **(B)** Butter 49 g/2500 kcal. **(C)** MUFA-rich diet **(D)** PUFA-rich diet. **(E)** High-cholesterol, low-fat diet. The SFA content was matched in diets **A** + **B**.	Five 4-week periods, separated by 4-week washout periods	No changes were evident in HDL after cheese consumption. LDL was lower in group **A** compared with group **B**, but higher than groups **C**, **D** and **E**. No significant differences were found in inflammation markers, blood pressure and insulin-glucose homeostasis.
Raziani et al. 2016 [64]	Denmark	Parallel	*n* = 139 (47 male; 92 female), risk of metabolic syndrome	18–70	3 arm: **(A)** 40 g equal parts regular-fat Danbo and cheddar cheese (REG). **(B)** Reduced-fat Danbo and cheddar cheese, 40 g, (RED). **(C)** Noncheese, carbohydrate control (CHO40 g/d)—90 g bread, 25 g jam.	12 weeks	No differences were evident on lipid profile between groups. In addition, Insulin, glucose, and triacylglycerol concentrations as well as blood pressure and waist circumference did not differ.
Thorning et al. 2015 [65]	Denmark	Crossover	*n* = 14 female, overweight, post-menopausal	45–68	3 arm: **(A)** 96–120 g of equal parts Dando and cheddar cheese per 8–10 MJ diet. **(B)** High fat meat—164 g per 10-MJ diet. **(C)** Non-dairy, low-fat control (CHO)—fruit (84 g), white bread, pasta and rice (58 g), marmalade (20 g), and cake, sweetened biscuits and chocolate (13 g) per 10-MJ diet.	Three 2-week periods, separated by 2-week washout periods	Group **A** caused higher levels of circulating HDL levels and apo A-I concentrations, and a lower apoB:apo A-I ratio compared to group **C**. Faecal fat excretion was also higher in group **A**. TC and LDL was similar across all groups.
Nilsen et al. 2015 [66]	Norway	Parallel	*n* = 153 (73 male; 80 female), normotensive and hypertensive	>18	3 arm: **(A)** 50 g/d Gamalost. **(B)** Norvegia 80 g/d. **(C)** Control—limited intake of Gamalost and Norvegia.	8 weeks	There were no changes in MetS (metabolic equivalents) factors between the intervention groups and control. Significant reductions were noted for TC in those with MetS in group **B**. Those in group **A** with high TC also showed significant decreases compared with control.
Soerensen et al. 2014 [67]	Denmark	Crossover	*n* = 15 males, healthy volunteers	18–50	3 arm: **(A)** 500 mg Ca/d-non-dairy control. **(B)** Semi-skimmed milk, 670 mL per 10 MJ. **(C)** Semi hard cheese, 120 g per 10 MJ (45% fat).	Three 2-week periods, separated by 2-week washout period	Significantly lower increases in TC and LDL were found in the group **B** and **C** compared with control. Faecal fat excretion also increased group **B** and **C** compared with control. No changes were found in blood pressure, high-density lipoprotein cholesterol, triglycerides and lipid ratios.
Hjerpsted et al. 2011 [68]	Denmark	Crossover	*n* = 49 (28 male; 21 female), healthy volunteers	22–69	Test-food amounts were dependent on participants’ energy levels. 2 arm: **(A)** 143 g/d (based on medium energy level) hard cheese “Samsø” (27 g fat/100 g). **(B)** Salted butter, 47 g/d (based on medium energy level).	Two 6-week periods, separated by 2-week washout period	Group **A** had significantly lower serum total, LDL and HDL cholesterol, and increased glucose concentrations compared with group **B**. Faecal fat excretion did not differ between groups.
Intorre et al. 2011 [69]	Italy	Crossover	*n* = 30 (11 male; 19 female), healthy volunteers	20–40	2 arm: **(A)** 150 g of hard cheese per week (milk from cows fed a grass and maize silage-based diet with 5% of linseed oil added). **(B)** Control, 150 g of hard cheese per week (from normal cow milk)	Two 4-week periods, separated by a 4-week washout	The blood lipid profile did not change after diet **A**. Although it led to higher levels of vitamin C and E and stearic acid in blood, while myristic acid and oxidised LDL concentrations were significantly lower.
Pintus et al. 2013 [70]	Italy	Crossover	*n* = 42 (19 male; 23 female), mildly hypercholesterolaemic	30–60	2 arm: **(A)** control, 90 g/d sheep cheese. **(B)** Sheep cheese, 90 g/d, naturally enriched with CLA	Two 3-week periods, separated by a 6-week washout	The findings confirmed an association between anandamide and adiposity. Diet **B** significantly increased the plasma levels of fatty acid hydrocyperoxidases and LDL decreased. However, no changes were detected in levels of inflammatory markers.
Sofi et al. 2010 [71]	Italy	Crossover	*n* = 10 (4 male; 6 female) healthy volunteers	30–65	2 arm: **(A)** 200 g/week (3 times a week) of pecorino cheese, naturally rich in CLA. **(B)** Placebo cheese—control, 200 g/week (3 times a week).	Two 10-week periods, separated by a 10-week washout period	Consumption of cheese naturally rich in CLA determined a significant reduction in some inflammatory parameters as well as some haemorheological, appearing to cause favourable biochemical changes of atherosclerotic markers, albeit limited. No significant effects on lipid profile were evident.
Nestel et al. 2005 [72]	Australia	Crossover	*n* = 19 (14 male; 5 female), overweight and mildly hypercholesterolaemic	Average— 56.3 ± 7.8	2 arm: **(A)** 120 g/d mature cheddar (40 g fat). **(B)** Similar amount of butter fat to group **A**, from preweighed portions of butter and one butter-rich muffin.	Two 4-week periods, separated by a 2-week washout period	Lipid values did not differ significantly between the group **A** and run-in periods, but TC and LDL were significantly higher with group **B**. Group **B** (butter) also raised total and LDL cholesterol significantly. This was not evident for cheese.
Biong et al. 2004 [73]	Norway	Crossover	*n* = 22 (9 male; 13 female), healthy volunteers	21–54	3 arm: **(A)** 150 g/d (per 8 MJ diet) Jarlsberg ‘Swiss-type’ cheese. **(B)** Butter, 52 g/d (per 8 MJ diet) + casein (as calcium caseinate). **(C)** Butter, 52 g/d (per 8 MJ diet) + egg white.	Three 3-week periods, separated by a 1-week washout periods	TC was significantly lower after diet **A** compared to diet **B**. While LDL was lower after diet **A**, this was not statistically significant. There were also no significant differences in HDL-cholesterol, triacylglycerols, apo A-I, apo B or lipoprotein (a), haemostatic variables and homocysteine between groups.
Karvonen et al. 2002 [74]	Finland	Crossover	*n* = 31 (17 male; 14 female), hyperlipidaemic	25–65	2 arm: **(A)** 65 g/d low-fat rapeseed oil-based cheese (11 g fat, of which 1 g was SFA). **(B)** Hard cheese, 65 g/d (15 g fat, of which 10 g was SFA).	Two 4-week periods, washout period not specified	Serum TC and LDL concentration was lower in group **A**, 2 and 4 weeks after use of rapeseed oil-based cheese compared to group **B** (control).

**Table 3:** Randomised controlled trials (RCT) demonstrating an effect of milk consumption on markers of metabolic health and cardiovascular disease (CVD) risk.

Author	Country	Study Design	Population	Age (Years)	Intervention	Duration	Main Findings
Lee et al. 2016 [78]	Korea	Parallel	*n* = 58 (29 male; 29 female), overweight and obese with metabolic syndrome	35–65	2 arm: **(A)** 400 mL per day (200 mL twice daily) of low-fat milk. **(B)** Control—maintain habitual diet.	6 weeks	No significant differences in body mass index, blood pressure, lipid profile and adiponectin levels, as well as levels of inflammatory markers, oxidative stress markers and atherogenic markers were found between groups.
Hidaka et al. 2012 [79]	Japan	Parallel	*n* = 14 (8 male; 6 female), healthy volunteers	Mean: 28.6 ± 6.0 S.D	2 arm: **(A)** 500 mL/d whole milk. **(B)** non-fat milk, 500 mL/d.	4 weeks	Group **B** showed lowering of plasma triglyceride (TG), phospholipid levels, TG level in HDL and increased plasma apolipoprotein (apo) C-III level. TG/cholesterol ratios in HDL and LDL also significantly decreased in group **B**. Whole milk consumption showed increases in plasma levels of apoC-III and apoE. C.
Rosado et al. 2011 [80]	Mexico	Parallel	*n* = 139 females, obese	25–45	3 arm: **(A)** 250 mL of low-fat milk, 3 times per day, and an energy-restricted diet (500 kcal/day). **(B)** Low-fat milk, 250 mL, with added micronutrients, 3 times per day, an energy-restricted diet (500 kcal/day). **(C)** Control—an energy-restricted diet (500 kcal/day) with no intake of milk.	16 weeks	Group **B** lost significantly more weight compared with group **A** + **C**. BMI and body fat changes were also significantly greater in the group **B** compared with group **A** + **C**. No differences were found between groups in glucose level, blood lipid profile, C-reactive protein level or blood pressure.
Venkatramanan et al. 2010 [81]	Canada	Crossover	*n* = 18 (11 male; 7 female) moderately overweight and borderline hyperlipidaemic	30–60	3 arm: **(A)** 1000 mL/d milk naturally enriched CLA. **(B)** Milk enriched with synthetic CLA, 1000 mL/d. **(C)** Control—1000 mL/d untreated milk.	Three 8-week periods separated by 4-week washout periods	Group **A** + **B** failed to alter plasma TC, LDL, HDL or triacylglycerol concentrations; body weight; or fat composition compared with the control group. CLA consumption did not significantly affect plasma ALT (alanine transaminase), TBIL (total bilirubin in plasma), CRP (C-reactive protein) or TNF-a (tumor necrosis factor) concentrations.
Faghih et al. 2009 [82]	Iran	Parallel	*n* = 100 females, premenopausal and overweight or obese	20–50	4 arm: **(A)** control—500 kcal/d deficit (500–600 mg/d dietary calcium). **(B)** Calcium-supplemented diet identical to control diet (800 mg/d of calcium carbonate). **(C)** Servings of low-fat milk, 220 mL, (1.5%) and 500 kcal/d deficit. **(D)** Three servings of calcium-fortified soy milk and 500 kcal/d deficit.	8 weeks	Body weight, BMI, waist circumference (WC), waist-to-hip ratio (WHR), body fat mass and percent body fat decreased significantly across all groups. The changes in WC and WHR were significantly higher in groups **C** and **D** compared to controls. Reductions in weight and BMI were significantly greater in the group **C** compared to controls. Lipid profiles were not analysed.
Gardner et al. 2007 [77]	USA	Crossover	*n* = 28 (6 male; 22 female), hypercholesteraemic	30–65	3 arm: 32 oz/d whole soy bean drink. **(B)** Soy protein isolate drink, 28 oz/d. **(C)** Dairy milk, 18.5 oz/d, (all volumes were standardised to yield 25 g protein/d.	Three 4-week periods, separated by 4-week washout periods	LDL was significantly lower after consuming soy milk in groups **A** + **B** compared to dairy milk (group **C**). No significant differences between groups were observed for HDL, triacylglycerols, insulin or glucose.
Barr et al. 2000 [83]	USA	Parallel	*n* = 200 (70 male; 130 female), healthy volunteers	55–85	2 arm: **(A)** three 8 oz/d of skimmed 1% milk. **(B)** Maintain habitual diet and consuming <1.5 dairy servings per day.	12 weeks	Similar decreases in blood pressure were apparent across both groups. TC, LDL and the ratio of TC:HDL remained unchanged. Triglyceride levels increased within the normal range in group **A**.

**Table 4:** Randomised controlled trials (RCT) demonstrating an effect of yoghurt consumption on markers of metabolic health and CVD risk.

Author	Country	Study Design	Population	Age (Years)	Intervention	Duration	Main Findings
**El Khoury et al. 2014** [84]	Canada	Crossover	*n* = 20 males, BMI 20–24.9 kg/m²	20–30	5 arm: **(A)** 250 g non-fat yoghurt—plain. **(B)** Non-fat yoghurt with honey—plain, 250 g. **(C)** Non-fat yoghurt, strawberry flavoured, 250 g. **(D)** Skimmed milk, 250 g. **(E)** Orange Juice, 250 g.	Postprandial	Pre-meal glucose responses were dose-dependent to increasing protein and decreasing sugars in dairy. Protein:carbohydrate ratio correlated negatively with pre-meal glucose due to improved efficacy of insulin action. Compared with treatment E, blood glucose was lower after dairy snack treatments, contribution of dairy products to post-meal glucose was independent of their protein:carbohydrate ratio
**Shab-Bidar et al. 2011** [85]	Iran	Parallel	*n* = 100 (43 male; 57 female), type 2 diabetes	29–67	2 arm: **(A)** 250 mL vitamin D3 fortified yoghurt drink, twice a day **(B)** 250 mL plain yoghurt fortified drink, twice a day.	12 weeks	Diet **A** showed significant improvement in fasting glucose, glycated haemoglobin (HbA1c), TAG, HDL cholesterol, endothelin-1, E-selectin and MMP-9 compared with the control (diet B).
**Sadrzadeh-Yeganeh et al. 2010** [86]	Iran	Parallel	*n* = 90 females, healthy volunteers	19–49	3 arm: **(A)** 300 g/d probiotic yoghurt. **(B)** Conventional yoghurt, 300 g/d. **(C)** Control, no consumption of fermented products	6 weeks	No significant difference in lipid profile within any group. No difference in TAG and LDL across the groups. There was a decrease in cholesterol in both group A + B compared with the control as well as a decrease in TC:HDL ratio. HDL increased in group A compared with the control.
**Ejtahed et al. 2010** [87]	Iran	Parallel	*n* = 60 (23 male; 37 female), type 2 diabetes	30–60	2 arm: **(A)** 300 g/d probiotic yoghurt. **(B)** Control, 300 g/d conventional yoghurt	6 weeks	Diet **A** caused a decrease in TC and LDL compared with the control. No significant changes from baseline were shown in TAG and HDL in diet A. The TC:HDL ratio and LDL:HDL ratio as atherogenic indices significantly decreased in after diet A compared with the control.
**Ataie-Jafari et al. 2009** [88]	Iran	Crossover	*n* = 14 (4 male; 10 female), mild to moderate hypercholesteraemic	40–64	2 arm: **(A)** 3 × 100 g/d probiotic yoghurt, *Lactobacillus acidophilus* and *Bifidobacterium lactis* **(B)** Three × 100 g/day control yoghurt. Both yoghurts contain 2.5% fat.	two 6-week periods, separated by a 4-week washout period	Consumption of diet **A** caused a significant decrease in serum TC compared with the control. No differences were reported for remaining blood lipids examined between the two diets.
**Kiessling et al. 2002** [89]	Germany	Crossover	*n* = 29 females, normo- and hypercholesterolaemic	19–56	2 arm: **(A)** 300 g/d control yoghurt *streptococcus thermophilus* and *L. lactis*. **(B)** Probiotic yoghurt, 300 g/d, enriched with *L. acidophilus* 145, *B. longum* 913 and 1% oligofructose.	6 weeks (all on control diet), followed by two 6-week periods, separated by a 9-day washout periods.	Serum TC and LDL concentrations were not influenced by diet A. The HDL concentration increased significantly after diet **A**. The ratio of LDL:HDL cholesterol decreased. Long-term consumption of 300 g yoghurt increased HDL and lead to desired improvement of LDL:HDL ratio in both diets.
**Rizkalla et al. 2000** [90]	France	Crossover	*n* = 24 males, healthy volunteers	20–60	2 arm: **(A)** 500 g/d fresh yoghurt w/ live bacterial cultures. **(B)** Heated yoghurt, 500 g/d	Two 15-day periods, separated by a 15-day washout period	No changes detected in fasting plasma glucose, insulin, fatty acid, TAG or cholesterol concentrations in both groups. Plasma butyrate was higher and plasma propionate tended to be higher in subjects without lactose malabsorption after diet **A** than **B**. Subjects with lactose malabsorption increased propionate production after fresh yoghurt consumption compared with baseline measures.
**Agerholm-Larson et al. 2000** [91]	Denmark	Parallel	*n* = 70 (20 male; 50 female), healthy volunteers	18–55	5 arm: **(A)** 450 mL/d yoghurt fermented with two strains of *Streptococcus thermophilus* and two strains of *Lactobacillus acidophilus.* **(B)** Placebo yoghurt, 450 mL/d, fermented with delta-acid-lactone. **(C)** Yoghurt, 450 mL/d, fermented with two strains of *Streptococcus thermophilus* and one strain of *Lactobacillus rhamnosus.* **(D)** Yoghurt, 450 mL/d, fermented with one strain of *Enterococcus faecium* and two strains of *Streptococcus thermophilus.* **(E)** Two placebo pills per day.	8 weeks	Comparing all 5 groups, no statistical effects on LDL were observed after consumption of diet D. After adjusting for small changes in body weight, LDL decreased by 8.4% and fibrinogen increased. This was significantly different from the control groups, **B** + **E**. After diets **A** + **D**, systolic blood pressure reduced significantly more compared to diet **C**.
**Anderson and Gilliland, 1999** [92]	USA	Study 1: single-blind, parallel. Study 2: double-blind, crossover.	Study 1: *n* = 29 (9 male; 20 female). Study 2: *n* = 40 (18 male; 22 female), all hypercholesterolaemic	49–55	**Study 1**: 2 arm: **(A1)** 200 g/d fermented milk yoghurt containing human *L. acidophilus* L1. **(B1)** Fermented milk yoghurt, 200 g/d, containing swine *L. acidophilus* strain ATCC 43121 **Study 2**: 2 arm: **(A2)** 200 g/d fermented milk yoghurt containing *L. acidophilus* L1 strain. (B) Fermented milk yoghurt, 200 g/d, without these active bacteria.	Study 1: 3 weeks Study 2: Two 4-week periods with a 2-week washout period	Study 1: Diet **A1** showed a significant 2.4% reduction in TC. LDL was lower after both treatment groups although not significant. HDL decreased significantly in both groups. Study 2: diet **A2** reduced TC in the 1st treatment period but not in the 2nd. A combined analysis of the two treatment study interventions demonstrated a significant reduction in TC.

## References

[1] Haug A., Høstmark A.T., Harstad O.M. (2007). Bovine milk in human nutrition—A review. Lipids Health Dis..

[2] Marangoni F., Pellegrino L., Verduci E., Ghiselli A., Bernabei R., Calvani R., Cetin I., Giampietro M., Perticone F., Piretta L. (2019). Cow’s milk consumption and health: A health professional’s guide. J. Am. Coll. Nutr..

[3] Rosqvist F., Smedman A., Lindmark-Månsson H., Paulsson M., Petrus P., Straniero S., Rudling M., Dahlman I., Risérus U. (2015). Potential role of milk fat globule membrane in modulating plasma lipoproteins, gene expression, and cholesterol metabolism in humans: A randomized study. Am. J. Clin. Nutr..

[4] Ross A.C., Taylor C.L., Yaktine A.L., Del Valle H.B., Institute of Medicine Committee to Review Dietary Reference Intakes for Calcium and Vitamin D (2011). The National Academies Collection: Reports funded by National Institutes of Health. Dietary Reference Intakes for Calcium and Vitamin D.

[5] Gueguen L., Pointillart A. (2000). The bioavailability of dietary calcium. J. Am. Coll. Nutr..

[6] Zhao Y., Martin B.R., Weaver C.M. (2005). Calcium bioavailability of calcium carbonate fortified soymilk is equivalent to cow’s milk in young women. J. Nutr..

[7] Fulgoni V.L., Keast D.R., Auestad N., Quann E.E. (2011). Nutrients from dairy foods are difficult to replace in diets of Americans: Food pattern modeling and an analyses of the National Health and Nutrition Examination Survey. Nutr. Res..

[8] Dror D.K., Allen L.H. (2014). Dairy product intake in children and adolescents in developed countries: Trends, nutritional contribution, and a review of association with health outcomes. Nutr. Rev..

[9] Vissers P.A., Streppel M.T., Feskens E.J., de Groot L.C. (2011). Contribution of dairy products to micronutrient intake in The Netherlands. J. Am. Coll. Nutr..

[10] Coudray B. (2011). Contribution of dairy products to micronutrient intake in France. J. Am. Coll. Nutr..

[11] Kalkwarf H.J., Khoury J.C., Lanphear B.P. (2003). Milk intake during childhood and adolescence, adult bone density, and osteoporotic fractures in US women. Am. J. Clin. Nutr..

[12] Ma D.F., Zheng W., Ding M., Zhang Y.M., Wang P.Y. (2013). Milk intake increases bone mineral content through inhibiting bone resorption: Meta-analysis of randomized controlled trials. e-SPEN J..

[13] Dror D.K. (2014). Dairy consumption and pre-school, school-age and adolescent obesity in developed countries: A systematic review and meta-analysis. Obes. Rev..

[14] Spence L.A., Cifelli C.J., Miller G.D. (2011). The Role of Dairy Products in Healthy Weight and Body Composition in Children and Adolescents. Curr. Nutr. Food. Sci..

[15] Brantsæter A.L., Olafsdottir A.S., Forsum E., Olsen S.F., Thorsdottir I. (2012). Does milk and dairy consumption during pregnancy influence fetal growth and infant birthweight? A systematic literature review. Food Nutr. Res..

[16] Geiker N.R.W., Mølgaard C., Iuliano S., Rizzoli R., Manios Y., van Loon L.J.C., Lecerf J.M., Moschonis G., Reginster J.Y., Givens I. (2020). Impact of whole dairy matrix on musculoskeletal health and aging–current knowledge and research gaps. Osteoporos. Int..

[17] Granic A., Hurst C., Dismore L., Aspray T., Stevenson E., Witham M.D., Sayer A.A., Robinson S. (2020). Milk for Skeletal Muscle Health and Sarcopenia in Older Adults: A Narrative Review. Clin. Interv. Aging.

[18] Thorning T.K., Raben A., Tholstrup T., Soedamah-Muthu S.S., Givens I., Astrup A. (2016). Milk and dairy products: Good or bad for human health? An assessment of the totality of scientific evidence. J. Food Nutr. Res..

[19] Food and Agricultural Organization of the United Nations Dairy Development in Asia. http://www.fao:3/i0588e/I0588E02.htm.

[20] Cifelli C.J., Houchins J.A., Demmer E., Fulgoni V.L. (2016). Increasing Plant Based Foods or Dairy Foods Differentially Affects Nutrient Intakes: Dietary Scenarios Using NHANES. Nutrients.

[21] Weinberger M.H. (1996). Salt Sensitivity of Blood Pressure in Humans. Hypertension.

[22] Strazzullo P., D’Elia L., Kandala N.-B., Cappuccio F.P. (2009). Salt intake, stroke, and cardiovascular disease: Meta-analysis of prospective studies. BMJ.

[23] Ni Mhurchu C., Capelin C., Dunford E.K., Webster J.L., Neal B.C., Jebb S.A. (2010). Sodium content of processed foods in the United Kingdom: Analysis of 44,000 foods purchased by 21,000 households. Am. J. Clin. Nutr..

[24] Anderson C.A., Appel L.J., Okuda N., Brown I.J., Chan Q., Zhao L., Ueshima H., Kesteloot H., Miura K., Curb J.D. (2010). Dietary sources of sodium in China, Japan, the United Kingdom, and the United States, women and men aged 40 to 59 years: The INTERMAP study. J. Am. Diet. Assoc..

[25] Sacks F.M., Svetkey L.P., Vollmer W.M., Appel L.J., Bray G.A., Harsha D., Obarzanek E., Conlin P.R., Miller E.R., Simons-Morton D.G. (2001). Effects on Blood Pressure of Reduced Dietary Sodium and the Dietary Approaches to Stop Hypertension (DASH) Diet. N. Engl. J. Med..

[26] Huth P.J., Fulgoni V.L., Keast D.R., Park K., Auestad N. (2013). Major food sources of calories, added sugars, and saturated fat and their contribution to essential nutrient intakes in the U.S. diet: Data from the National Health and Nutrition Examination Survey (2003–2006). Nutr. J..

[27] Feeney E.L., Nugent A.P., Mc Nulty B., Walton J., Flynn A., Gibney E.R. (2016). An overview of the contribution of dairy and cheese intakes to nutrient intakes in the Irish diet: Results from the National Adult Nutrition Survey. Br. J. Nutr..

[28] Scientific Advisory Committee on Nutrition Saturated Fats and Health. https://assets.publishing.service.gov.uk/government/uploads/system/uploads/attachment_data/file/814995/SACN_report_on_saturated_fat_and_health.pdf.

[29] Department of Health, Ireland (2016). Your Guide to Healthy Eating Using the Food Pyramid. https://www.hse.ie/eng/about/who/healthwellbeing/our-priority-programmes/heal/food-pyramid-images/food-pyramid-simple-version.pdf.

[30] De Oliveira M.C.O., Mozaffarian D., Kromhout D., Bertoni A.G., Sibley C.T., Jacobs D.R., Nettleton J.A. (2012). Dietary intake of saturated fat by food source and incident cardiovascular disease: The Multi-Ethnic Study of Atherosclerosis. Am. J. Clin. Nutr..

[31] O’Sullivan T.A., Hafekost K., Mitrou F., Lawrence D. (2013). Food sources of saturated fat and the association with mortality: A meta-analysis. Am. J. Public Health.

[32] Feeney E.L., Barron R., Dible V., Hamilton Z., Power Y., Tanner L., Flynn C., Bouchier P., Beresford T., Noronha N. (2018). Dairy matrix effects: Response to consumption of dairy fat differs when eaten within the cheese matrix—A randomized controlled trial. Am. J. Clin. Nutr..

[33] Chen M., Pan A., Malik V.S., Hu F.B. (2012). Effects of dairy intake on body weight and fat: A meta-analysis of randomized controlled trials. Am. J. Clin. Nutr..

[34] Menotti A., Keys A., Aravanis C., Blackburn H., Dontas A., Fidanza F., Karvonen M.J., Kromhout D., Nedeljkovic S., Nissinen A. (1989). Seven Countries Study. First 20-Year Mortality Data in 12 Cohorts of Six Countries. Ann. Med..

[35] Kromhout D., Bloemberg B., Feskens E., Menotti A., Nissinen A., Seven Countries Study Group (2000). Saturated fat, vitamin C and smoking predict long-term population all-cause mortality rates in the Seven Countries Study. Int. J. Epidemiol..

[36] Jacobson T.A., Maki K.C., Orringer C.E., Jones P.H., Kris-Etherton P., Sikand G., La Forge R., Daniels S.R., Wilson D.P., Morris P.B. (2015). National Lipid Association recommendations for patient-centered management of dyslipidemia: Part 2. J. Clin. Lipidol..

[37] Eckel R.H., Jakicic J.M., Ard J.D., de Jesus J.M., Miller N.H., Hubbard V.S., Lee I.-M., Lichtenstein A.H., Loria C.M., Millen B.E. (2014). 2013 AHA/ACC guideline on lifestyle management to reduce cardiovascular risk: A report of the American College of Cardiology/American Heart Association Task Force on Practice Guidelines. J. Am. Coll. Cardiol..

[38] USDA Scientific Report of the 2015 Dietary Guidelines Advisory Committee: Advisory Report to the Secretary of Health and Human Services and the Secretary of Agriculture. https://health.gov/sites/default/files/2019-09/Scientific-Report-of-the-2015-Dietary-Guidelines-Advisory-Committee.pdf.

[39] De Souza R.J., Mente A., Maroleanu A., Cozma A.I., Ha V., Kishibe T., Uleryk E., Budylowski P., Schünemann H., Beyene J. (2015). Intake of saturated and trans unsaturated fatty acids and risk of all cause mortality, cardiovascular disease, and type 2 diabetes: Systematic review and meta-analysis of observational studies. BMJ.

[40] Drouin-Chartier J.-P., Brassard D., Tessier-Grenier M., Côté J.A., Labonté M.-È., Desroches S., Couture P., Lamarche B. (2016). Systematic review of the association between dairy product consumption and risk of cardiovascular-related clinical outcomes. Adv. Nutr..

[41] Alexander D.D., Bylsma L.C., Vargas A.J., Cohen S.S., Doucette A., Mohamed M., Irvin S.R., Miller P.E., Watson H., Fryzek J.P. (2016). Dairy consumption and CVD: A systematic review and meta-analysis. Br. J. Nutr..

[42] Antoni R., Griffin B. (2018). Draft reports from the UK’s Scientific Advisory Committee on Nutrition and World Health Organization concur in endorsing the dietary guideline to restrict intake of saturated fat. Nutr. Bull..

[43] Astrup A., Bertram H.C., Bonjour J.-P., De Groot L.C., de Oliveira Otto M.C., Feeney E.L., Garg M.L., Givens I., Kok F.J., Krauss R.M. (2019). WHO draft guidelines on dietary saturated and trans fatty acids: Time for a new approach?. BMJ.

[44] Dehghan M., Mente A., Rangarajan S., Sheridan P., Mohan V., Iqbal R., Gupta R., Lear S., Wentzel-Viljoen E., Avezum A. (2018). Association of dairy intake with cardiovascular disease and mortality in 21 countries from five continents (PURE): A prospective cohort study. Lancet.

[45] Fontecha J., Calvo M.V., Juarez M., Gil A., Martínez-Vizcaino V. (2019). Milk and dairy product consumption and cardiovascular diseases: An overview of systematic reviews and meta-analyses. Adv. Nutr..

[46] Zemel M.B., Sun X., Sobhani T., Wilson B. (2010). Effects of dairy compared with soy on oxidative and inflammatory stress in overweight and obese subjects. Am. J. Clin. Nutr..

[47] Vasilopoulou D., Markey O., Kliem K.E., Fagan C.C., Grandison A.S., Humphries D.J., Todd S., Jackson K.G., Givens D.I., Lovegrove J.A. (2020). Reformulation initiative for partial replacement of saturated with unsaturated fats in dairy foods attenuates the increase in LDL cholesterol and improves flow-mediated dilatation compared with conventional dairy: The randomized, controlled REplacement of SaturatEd fat in dairy on Total cholesterol (RESET) study. Am. J. Clin. Nutr..

[48] Drouin-Chartier J.-P., Li Y., Ardisson Korat A.V., Ding M., Lamarche B., Manson J.E., Rimm E.B., Willett W.C., Hu F.B. (2019). Changes in dairy product consumption and risk of type 2 diabetes: Results from 3 large prospective cohorts of US men and women. Am. J. Clin. Nutr..

[49] Markey O., Vasilopoulou D., Kliem K.E., Koulman A., Fagan C.C., Summerhill K., Wang L.Y., Grandison A.S., Humphries D.J., Todd S. (2017). Plasma phospholipid fatty acid profile confirms compliance to a novel saturated fat-reduced, monounsaturated fat-enriched dairy product intervention in adults at moderate cardiovascular risk: A randomized controlled trial. Nutr. J..

[50] Benatar J.R., Jones E., White H., Stewart R.A. (2014). A randomized trial evaluating the effects of change in dairy food consumption on cardio-metabolic risk factors. Eur. J. Prev. Cardiol..

[51] Nestel P.J., Mellett N., Pally S., Wong G., Barlow C.K., Croft K., Mori T.A., Meikle P.J. (2013). Effects of low-fat or full-fat fermented and non-fermented dairy foods on selected cardiovascular biomarkers in overweight adults. Br. J. Nutr..

[52] Crichton G.E., Howe P.R., Buckley J.D., Coates A.M., Murphy K.J. (2012). Dairy consumption and cardiometabolic health: Outcomes of a 12-month crossover trial. Nutr. Metab..

[53] Palacios C., Bertrán J.J., Ríos R.E., Soltero S. (2011). No effects of low and high consumption of dairy products and calcium supplements on body composition and serum lipids in Puerto Rican obese adults. Nutrition.

[54] Stancliffe R.A., Thorpe T., Zemel M.B. (2011). Dairy attentuates oxidative and inflammatory stress in metabolic syndrome. Am. J. Clin. Nutr..

[55] Van Meijl L.E., Mensink R.P. (2010). Effects of low-fat dairy consumption on markers of low-grade systemic inflammation and endothelial function in overweight and obese subjects: An intervention study. Br. J. Nutr..

[56] Wennersberg M.H., Smedman A., Turpeinen A.M., Retterstøl K., Tengblad S., Lipre E., Aro A., Mutanen P., Seljeflot I., Basu S. (2009). Dairy products and metabolic effects in overweight men and women: Results from a 6-mo intervention study. Am. J. Clin. Nutr..

[57] Van Meijl L.E., Mensink R.P. (2010). Low-fat dairy consumption reduces systolic blood pressure, but does not improve other metabolic risk parameters in overweight and obese subjects. Nutr. Metab. Cardiovasc. Dis..

[58] Tricon S., Burdge G.C., Jones E.L., Russell J.J., El-Khazen S., Moretti E., Hall W.L., Gerry A.B., Leake D.S., Grimble R.F. (2006). Effects of dairy products naturally enriched with cis-9, trans-11 conjugated linoleic acid on the blood lipid profile in healthy middle-aged men. Am. J. Clin. Nutr..

[59] Zemel M.B., Richards J., Milstead A., Campbell P. (2005). Effects of calcium and dairy on body composition and weight loss in African-American adults. Obes. Res..

[60] Tholstrup T., Høy C.-E., Andersen L.N., Christensen R.D., Sandström B. (2004). Does fat in milk, butter and cheese affect blood lipids and cholesterol differently?. J. Am. Coll. Nutr..

[61] Brassard D., Tessier-Grenier M., Allaire J., Rajendiran E., She Y., Ramprasath V., Gigleux I., Talbot D., Levy E., Tremblay A. (2017). Comparison of the impact of SFAs from cheese and butter on cardiometabolic risk factors: A randomized controlled trial. Am. J. Clin. Nutr..

[62] Thorning T.K., Bertram H.C., Bonjour J.-P., De Groot L., Dupont D., Feeney E., Ipsen R., Lecerf J.M., Mackie A., McKinley M.C. (2017). Whole dairy matrix or single nutrients in assessment of health effects: Current evidence and knowledge gaps. Am. J. Clin. Nutr..

[63] Limongi F., Noale M., Marseglia A., Gesmundo A., Mele M., Banni S., Crepaldi G., Maggi S. (2018). Impact of cheese rich in Conjugated Linoleic Acid on low density lipoproteins cholesterol: Dietary Intervention in Older People (CLADIS Study). J. Food. Nutr. Res..

[64] Raziani F., Tholstrup T., Kristensen M.D., Svanegaard M.L., Ritz C., Astrup A., Raben A. (2016). High intake of regular-fat cheese compared with reduced-fat cheese does not affect LDL cholesterol or risk markers of the metabolic syndrome: A randomized controlled trial. Am. J. Clin. Nutr..

[65] Thorning T.K., Raziani F., Bendsen N.T., Astrup A., Tholstrup T., Raben A. (2015). Diets with high-fat cheese, high-fat meat, or carbohydrate on cardiovascular risk markers in overweight postmenopausal women: A randomized crossover trial. Am. J. Clin. Nutr..

[66] Nilsen R., Høstmark A.T., Haug A., Skeie S. (2015). Effect of a high intake of cheese on cholesterol and metabolic syndrome: Results of a randomized trial. Food. Nutr. Res..

[67] Soerensen K.V., Thorning T.K., Astrup A., Kristensen M., Lorenzen J.K. (2014). Effect of dairy calcium from cheese and milk on fecal fat excretion, blood lipids, and appetite in young men. Am. J. Clin. Nutr..

[68] Hjerpsted J., Leedo E., Tholstrup T. (2011). Cheese intake in large amounts lowers LDL-cholesterol concentrations compared with butter intake of equal fat content. Am. J. Clin. Nutr..

[69] Intorre F., Foddai M.S., Azzini E., Martin B., Montel M.-C., Catasta G., Toti E., Finotti E., Palomba L., Venneria E. (2011). Differential effect of cheese fatty acid composition on blood lipid profile and redox status in normolipidemic volunteers: A pilot study. Int. J. Food. Sci. Nutr..

[70] Pintus S., Murru E., Carta G., Cordeddu L., Batetta B., Accossu S., Pistis D., Uda S., Ghiani M.E., Mele M. (2013). Sheep cheese naturally enriched in α-linolenic, conjugated linoleic and vaccenic acids improves the lipid profile and reduces anandamide in the plasma of hypercholesterolaemic subjects. Br. J. Nutr..

[71] Sofi F., Buccioni A., Cesari F., Gori A.M., Minieri S., Mannini L., Casini A., Gensini G.F., Abbate R., Antongiovanni M. (2010). Effects of a dairy product (pecorino cheese) naturally rich in cis-9, trans-11 conjugated linoleic acid on lipid, inflammatory and haemorheological variables: A dietary intervention study. Nutr. Metab. Cardiovasc. Dis..

[72] Nestel P., Chronopulos A., Cehun M. (2005). Dairy fat in cheese raises LDL cholesterol less than that in butter in mildly hypercholesterolaemic subjects. Eur. J. Clin. Nutr..

[73] Biong A.S., Müller H., Seljeflot I., Veierød M.B., Pedersen J.I. (2004). A comparison of the effects of cheese and butter on serum lipids, haemostatic variables and homocysteine. Br. J. Nutr..

[74] Karvonen H., Tapola N., Uusitupa M., Sarkkinen E. (2002). The effect of vegetable oil-based cheese on serum total and lipoprotein lipids. Eur. J. Clin. Nutr..

[75] Drouin-Chartier J.-P., Tremblay A.J., Maltais-Giguère J., Charest A., Guinot L., Rioux L.-E., Labrie S., Britten M., Lamarche B., Turgeon S.L. (2017). Differential impact of the cheese matrix on the postprandial lipid response: A randomized, crossover, controlled trial. Am. J. Clin. Nutr..

[76] Hansson P., Holven K.B., Øyri L.K., Brekke H.K., Biong A.S., Gjevestad G.O., Raza G.S., Herzig K.-H., Thoresen M., Ulven S.M. (2019). Meals with similar fat content from different dairy products induce different postprandial triglyceride responses in healthy adults: A randomized controlled cross-over trial. J. Nutr..

[77] Gardner C.D., Messina M., Kiazand A., Morris J.L., Franke A.A. (2007). Effect of Two Types of Soy Milk and Dairy Milk on Plasma Lipids in Hypercholesterolemic Adults: A Randomized Trial. J. Am. Coll. Nutr..

[78] Lee Y.J., Seo J.A., Yoon T., Seo I., Lee J.H., Im D., Lee J.H., Bahn K.-N., Ham H.S., Jeong S.A. (2016). Effects of low-fat milk consumption on metabolic and atherogenic biomarkers in Korean adults with the metabolic syndrome: A randomised controlled trial. J. Hum. Nutr Diet..

[79] Hidaka H., Takiwaki M., Yamashita M., Kawasaki K., Sugano M., Honda T. (2012). Consumption of nonfat milk results in a less atherogenic lipoprotein profile: A pilot study. Ann. Nutr. Metab..

[80] Rosado J.L., Garcia O.P., Ronquillo D., Hervert-Hernández D., Caamaño M.D.C., Martínez G., Gutiérrez J., García S. (2011). Intake of milk with added micronutrients increases the effectiveness of an energy-restricted diet to reduce body weight: A randomized controlled clinical trial in Mexican women. J. Am. Diet. Assoc..

[81] Venkatramanan S., Joseph S.V., Chouinard P.Y., Jacques H., Farnworth E.R., Jones P.J. (2010). Milk enriched with conjugated linoleic acid fails to alter blood lipids or body composition in moderately overweight, borderline hyperlipidemic individuals. J. Am. Coll. Nutr..

[82] Faghih S.H., Abadi A.R., Hedayati M., Kimiagar S.M. (2009). Comparison of the effects of cows’ milk, fortified soy milk, and calcium supplement on weight and fat loss in premenopausal overweight and obese women. Nutr. Metab. Cardiovasc. Dis..

[83] Barr S.I., McCarron D.A., Heaney R.P., Dawson-Hughes B., Berga S.L., Stern J.S., Oparil S. (2000). Effects of increased consumption of fluid milk on energy and nutrient intake, body weight, and cardiovascular risk factors in healthy older adults. J. Am. Diet. Assoc..

[84] El Khoury D., Brown P., Smith G., Berengut S., Panahi S., Kubant R., Anderson G.H. (2014). Increasing the protein to carbohydrate ratio in yogurts consumed as a snack reduces post-consumption glycemia independent of insulin. Clin. Nutr..

[85] Shab-Bidar S., Neyestani T.R., Djazayery A., Eshraghian M.-R., Houshiarrad A., Gharavi A., Kalayi A., Shariatzadeh N., Zahedirad M., Khalaji N. (2011). Regular consumption of vitamin D-fortified yogurt drink (Doogh) improved endothelial biomarkers in subjects with type 2 diabetes: A randomized double-blind clinical trial. BMC Med..

[86] Sadrzadeh-Yeganeh H., Elmadfa I., Djazayery A., Jalali M., Heshmat R., Chamary M. (2010). The effects of probiotic and conventional yoghurt on lipid profile in women. Br. J. Nutr..

[87] Ejtahed H., Mohtadi-Nia J., Homayouni-Rad A., Niafar M., Asghari-Jafarabadi M., Mofid V., Akbarian-Moghari A. (2011). Effect of probiotic yogurt containing Lactobacillus acidophilus and Bifidobacterium lactis on lipid profile in individuals with type 2 diabetes mellitus. J. Dairy Sci..

[88] Ataie-Jafari A., Larijani B., Majd H.A., Tahbaz F. (2009). Cholesterol-lowering effect of probiotic yogurt in comparison with ordinary yogurt in mildly to moderately hypercholesterolemic subjects. Ann. Nutr. Metab..

[89] Kiessling G., Schneider J., Jahreis G. (2002). Long-term consumption of fermented dairy products over 6 months increases HDL cholesterol. Eur. J. Clin. Nutr..

[90] Rizkalla S.W., Luo J., Kabir M., Chevalier A., Pacher N., Slama G. (2000). Chronic consumption of fresh but not heated yogurt improves breath-hydrogen status and short-chain fatty acid profiles: A controlled study in healthy men with or without lactose maldigestion. Am. J. Clin. Nutr..

[91] Agerholm-Larsen L., Raben A., Haulrik N., Hansen A., Manders M., Astrup A. (2000). Effect of 8 week intake of probiotic milk products on risk factors for cardiovascular diseases. Eur. J. Clin. Nutr..

[92] Anderson J.W., Gilliland S.E. (1999). Effect of fermented milk (yogurt) containing Lactobacillus acidophilus L1 on serum cholesterol in hypercholesterolemic humans. J. Am. Coll. Nutr..

[93] Feeney E.L., McKinley M.C. (2020). The dairy food matrix: What it is and what it does. Milk and Dairy Foods: Their Functionality in Human Health and Disease.

[94] Ayala-Bribiesca E., Lussier M., Chabot D., Turgeon S.L., Britten M. (2016). Effect of calcium enrichment of Cheddar cheese on its structure, in vitro digestion and lipid bioaccessibility. Int. Dairy J..

[95] Lorenzen J.K., Nielsen S., Holst J.J., Tetens I., Rehfeld J.F., Astrup A. (2007). Effect of dairy calcium or supplementary calcium intake on postprandial fat metabolism, appetite, and subsequent energy intake. Am. J. Clin. Nutr..

[96] Ditscheid B., Keller S., Jahreis G. (2005). Cholesterol metabolism is affected by calcium phosphate supplementation in humans. J. Nutr..

[97] Antonopoulou S., Semidalas C.E., Koussissis S., Demopoulos C.A. (1996). Platelet-activating factor (PAF) antagonists in foods: A study of lipids with PAF or anti-PAF-like activity in cow’s milk and yogurt. J. Agric. Food Chem..

[98] Poutzalis S., Anastasiadou A., Nasopoulou C., Megalemou K., Sioriki E., Zabetakis I. (2016). Evaluation of the in vitro anti-atherogenic activities of goat milk and goat dairy products. Dairy Sci. Technol..

[99] Meisel H. (2001). Bioactive peptides from milk proteins: A perspective for consumers and producers. Aust. J. Dairy Technol..

[100] Gobbetti M., Stepaniak L., De Angelis M., Corsetti A., Di Cagno R. (2002). Latent bioactive peptides in milk proteins: Proteolytic activation and significance in dairy processing. Crit. Rev. Food Sci. Nutr..

[101] FitzGerald R.J., Meisel H. (2000). Milk protein-derived peptide inhibitors of angiotensin-I-converting enzyme. Br. J. Nutr..

[102] Hirota T., Ohki K., Kawagishi R., Kajimoto Y., Mizuno S., Nakamura Y., Kitakaze M. (2007). Casein hydrolysate containing the Antihypertensive Tripeptides Val-Pro-Pro and Ile-Pro-Pro improves vascular endothelial function independent of Blood Pressure–Lowering Effects: Contribution of the inhibitory action of Angiotensin-Converting enzyme. Hypertens. Res..

[103] Uenishi H., Kabuki T., Seto Y., Serizawa A., Nakajima H. (2012). Isolation and identification of casein-derived dipeptidyl-peptidase 4 (DPP-4)-inhibitory peptide LPQNIPPL from gouda-type cheese and its effect on plasma glucose in rats. Int. Dairy J..

[104] Gupta A., Mann B., Kumar R., Sangwan R.B. (2013). ACE-inhibitory activity of cheddar cheeses made with adjunct cultures at different stages of ripening. Adv. Dairy. Res..

[105] Gómez-Ruiz J.Á., Ramos M., Recio I. (2002). Angiotensin-converting enzyme-inhibitory peptides in Manchego cheeses manufactured with different starter cultures. Int. Dairy J..

[106] Lordan R., Zabetakis I. (2017). Invited review: The anti-inflammatory properties of dairy lipids. J. Dairy Sci..

[107] Norris G.H., Milard M., Michalski M.-C., Blesso C.N. (2019). Protective properties of milk sphingomyelin against dysfunctional lipid metabolism, gut dysbiosis, and inflammation. J. Nutr. Biochem..

[108] Millar C.L., Jiang C., Norris G.H., Garcia C., Seibel S., Anto L., Lee J.-Y., Blesso C.N. (2020). Cow’s milk polar lipids reduce atherogenic lipoprotein cholesterol, modulate gut microbiota and attenuate atherosclerosis development in LDL-receptor knockout mice fed a Western-type diet. J. Nutr. Biochem..

[109] Singh H. (2006). The milk fat globule membrane—A biophysical system for food applications. Curr. Opin. Colloid Interface Sci..

[110] Vanderghem C., Bodson P., Danthine S., Paquot M., Deroanne C., Blecker C. (2010). Milk fat globule membrane and buttermilks: From composition to valorization. Biotechnol. Agron. Soc. Environ..

[111] Demmer E., Van Loan M.D., Rivera N., Rogers T.S., Gertz E.R., German J.B., Smilowitz J.T., Zivkovic A.M. (2016). Addition of a dairy fraction rich in milk fat globule membrane to a high-saturated fat meal reduces the postprandial insulinaemic and inflammatory response in overweight and obese adults. J. Nutr. Sci..

[112] Burdge G.C., Calder P.C. (2005). Plasma cytokine response during the postprandial period: A potential causal process in vascular disease?. Br. J. Nutr..

[113] De Goede J., Geleijnse J.M., Ding E.L., Soedamah-Muthu S.S. (2015). Effect of cheese consumption on blood lipids: A systematic review and meta-analysis of randomized controlled trials. Nutr. Rev..

[114] Hjerpsted J.B. (2013). Cheese and Cardiovascular Health: Evidence from Observational, Intervention and Explorative Studies.

[115] Hjerpsted J., Tholstrup T. (2016). Cheese and cardiovascular disease risk: A review of the evidence and discussion of possible mechanisms. Crit. Rev. Food. Sci. Nutr..

[116] Feeney E.L., O’Sullivan A., Nugent A.P., McNulty B., Walton J., Flynn A., Gibney E.R. (2017). Patterns of dairy food intake, body composition and markers of metabolic health in Ireland: Results from the National Adult Nutrition Survey. Nutr. Diabetes.

[117] Rebholz C.M., Appel L.J. (2018). Health effects of dietary patterns: Critically important but vastly understudied. Am. J. Clin. Nutr..

[118] Markey O., Vasilopoulou D., Givens D.I., Lovegrove J.A. (2014). Dairy and cardiovascular health: Friend or foe?. Nutr. Bull..

[119] Raziani F., Ebrahimi P., Engelsen S.B., Astrup A., Raben A., Tholstrup T. (2018). Consumption of regular-fat vs reduced-fat cheese reveals gender-specific changes in LDL particle size—A randomized controlled trial. Nutr. Metab..

